# Partial Obstruction of Ventricular Catheters Affects Performance in a New Catheter Obstruction Model of Hydrocephalus

**DOI:** 10.3390/children9101453

**Published:** 2022-09-23

**Authors:** Seunghyun Lee, Michael Vinzani, Bianca Romero, Alvin Y. Chan, Leandro Castañeyra-Ruiz, Michael Muhonen

**Affiliations:** 1 CHOC Children’s Research Institute, 1201 W. La Veta Avenue, Orange, CA 92868, USA; 2 College of Medicine, Medical University of South Carolina, Charleston, SC 29425, USA; 3 CHOC Children’s Neuroscience Institute, 1201 W. La Veta Avenue, Orange, CA 92868, USA; 4 Department of Pediatric Neurosurgery, CHOC Children’s Hospital, 505 S Main St., Orange, CA 92868, USA

**Keywords:** hydrocephalus, shunt failure, catheter occlusion, ventricular phantom, flow/pressure performance

## Abstract

Objective: One of the major causes of cerebral ventricular shunt failure is proximal catheter occlusion. We describe a novel ventricular cerebrospinal fluid (CSF) flow replicating system that assesses pressure and flow responses to varying degrees of catheter occlusion. Methods: Ventricular catheter performance was assessed during conditions of partial and complete occlusion. The catheters were placed into a three-dimensionally-printed phantom ventricular replicating system. Artificial CSF was pumped through the ventricular system at a constant rate of 1 mL/min to mimic CSF flow, with the proximal end of the catheter in the phantom ventricle. Pressure transducer and flow rate sensors were used to measure intra-phantom pressure, outflow pressure, and CSF flow rates. The catheters were also inserted into silicone tubing and pressure was measured in the same manner for comparison with the phantom. Results: Pressure measured in the ventricle phantom did not change when the outflow of the ventricular catheter was partially occluded. However, the intraventricular phantom pressure significantly increased when the outflow catheter was 100% occluded. The flow through the catheter showed no significant difference in rate with any degree of partial occlusion of the catheter. At the distal end of the partially occluded catheters, there was less pressure compared with the nonoccluded catheters. This difference in pressure in partially occluded catheters correlated with the percentage of catheter hole occlusion. Conclusions: Our model mimics the physiological dynamics of the CSF flow in partially and completely obstructed ventricular catheters. We found that partial occlusion of the catheter had no effect on the CSF flow rate, but did reduce outflow pressure from the catheter.

## 1. Introduction

Hydrocephalus affects approximately 1 in 1100 children in the United States and is characterized by enlargement of ventricles due to impairment of cerebrospinal fluid (CSF) circulation [1,2]. The condition is often associated with elevated intracranial pressure (ICP), which can result in compression of vital structures (e.g., brain herniation syndromes) and hinder proper development [3]. The primary treatment of hydrocephalus is CSF diversion through a shunting system. Ventricular shunts divert CSF to an extra-cranial cavity, such as the peritoneum (i.e., ventriculoperitoneal shunt). However, pediatric CSF shunts frequently malfunction, with a 30–40% failure rate during the first year, 50% within the first two years, and up to 85% within 15 years [4,5]. One of the major causes of failure is occlusion of the ventricular catheter, accounting for over one-half of all pediatric shunt failures [6,7].

Efforts to avoid shunt obstruction have focused on modifying cell adhesion properties and immuno-activating the ventricular catheters to prevent cellular attachment [8]. However, these approaches may not prevent the immune response cascade induced by the placement of the catheter, which can result in persistent cellular blockage [9]. The detailed cellular mechanisms that underly catheter obstruction are somewhat unknown, though astrocytes and choroid plexus are thought to be primary drivers. The goal of this study was to assess the physiologic pressure and flow responses of partially and fully occluded ventricular catheters in a novel phantom ventricular system.

## 2. Methods

### 2.1. Catheter Occlusion Assay

The proximal portions of ventricular catheters (barium striped silicone catheter, Medtronic, Inc., Dublin, Ireland) were cut to ~4 cm length under sterile conditions. Epoxy resin was used to occlude the catheters, prior to testing their hydraulic properties with varying percentages of catheter hole occlusion. The catheters were subdivided into seven groups by the percentage of holes occluded in the proximal catheter: 0%, 20%, 40%, 60%, 80%, 95%, and 100%. A metal guide was inserted prior to applying the epoxy resin to the proximal holes to prevent unintentional clogging of the catheter lumen. All experiments were carried out after the resin was cured.

### 2.2. 3D Printing of the Ventricle Phantom

The ventricle phantom was printed by a 3D printer (Form 3B, Formlabs, Somerville, MA, USA) using flexible resin (Elastic 50A, Formlabs, Somerville, MA, USA). After printing, the model was washed for 20 min in a solvent of isopropyl alcohol (IPA) to remove uncured resin from the surface. Ultraviolet optical curing was performed for 20 min at 60 °C. The post-curing protocol ensured that the output structure had optimal mechanical and functional properties to operate as designed. The phantom had a volume of 150 mL, which was designed to mimic the expanded ventricles of a child with hydrocephalus.

### 2.3. Assessment of Catheter Pressure and Flow

**Figure 1:**
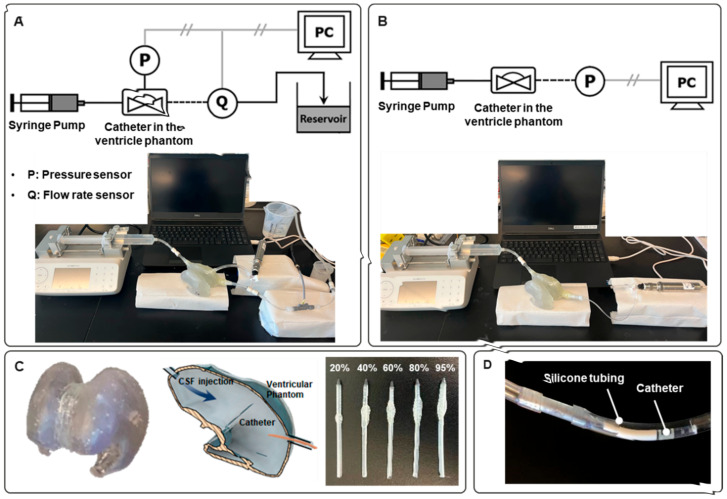
Experimental setup of the catheter performance system. A syringe pump (Fusion 200-X, Chemyx, Inc., Stafford, TX, USA) injecting artificial CSF at 1 mL/min was used as the fluid source. (**A**) Commercial pressure transducer (PX409-100 GUSBH, Omega, Inc., Biel/Bienne, Switzerland) and flow rate sensor (SLF3S-0600F, Sensirion, Inc., Stäfa, Switzerland) were used for measurement of internal pressure of the phantom and CSF flow rate through the catheter, respectively. (**B**) Experimental setup for pressure measurement in the catheter. (**C**) The 3D-printed ventricular phantom; actual printed part (**left**). The phantom was designed by Fusion 360 software and fabricated by SLA (Stereolithography) 3D printer (Form 3B, Formlabs) using elastic resin (Elastic 50A, Formlabs). Cross-sectional schematic of the phantom with the inserted catheter (middle). Catheters obstructed by epoxy resin by 20%, 40%, 60%, 80%, and 95% (from **left** to **right**). (**D**) A catheter was inserted into the silicon tubing instead of the phantom.

Bench-top testing was conducted to measure the hydraulic characteristics of the catheters, including pressure changes within the phantom model ventricle and the distal end of the ventricular catheter and CSF flow rate (Figure 1). The bench-top setup consisted of the ventricular catheter (barium striped silicone catheter, Medtronic, Inc., Dublin, Ireland), 3D printed ventricle phantom, syringe pump (Fusion 200-X, Chemyx, Inc., Stafford, TX, USA), a pressure transducer (PX409-100 GUSBH, Omega, Inc., Biel/Bienne, Switzerland), and flowrate sensor (SLF3S-0600F, Sensirion, Inc., Stäfa, Switzerland). The sample ventricular catheter was inserted into the phantom to replicate the conditions of CSF circulating through a shunted ventricle. As a control, and to determine if the phantom model influenced the resting state, the pressure was also measured after insertion of the catheters into silicone tubing, rather than directly into the phantom model (Figure 1D). A syringe pump filled with artificial CSF (LRE-S-LSG-1000-1, EcocyteShop) was set to deliver a flow of 1 mL/min, as verified with a calibrated flowmeter. A pressure transducer, with 0.001 psi resolution, was used to measure pressure in the phantom as well as at the distal end of the ventricular catheter. All data were recorded and collected in real-time.

### 2.4. Statistical Analysis

For flow and pressure quantification, 20 values were obtained per second, and each experiment consisted of a total of 240 s. Data sets are expressed as mean ± SEM. The Friedman test was used to determine whether differences between groups were statistically significant at a value of *p* < 0.05. All analyses were conducted using Prism 9 (GraphPad Software).

## 3. Results

### Catheter Performance under Obstructive Conditions

**Figure 2:**
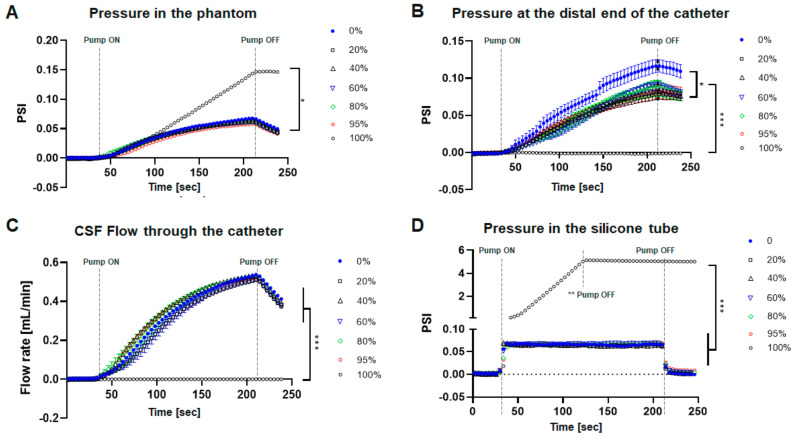
Catheter performance after epoxy obstruction. (**A**) Intraventricular pressure associated with barium striped silicone catheter malfunction due to epoxy resin obstruction. No significant differences were found between partially obstructed catheters and patent catheters. Significant differences were only found in 100% obstructed when compared with the rest of the conditions (*** *p* < 0.001). (**B**) Pressure measured at the distal end of the catheter obstructed by epoxy resin. Significant differences were found between 0% epoxy-obstructed catheters (* *p* < 0.05) and all the partially obstructed catheters, indicating that partial obstruction significantly reduced the outflow pressure of the catheter even though only 20% of the catheter holes were obstructed; 100% obstructed catheters significantly reduced the outflow pressure to 0 (*** *p* < 0.001). (**C**) The flow rate of CSF in the obstructed catheter. No significant differences were found according to the change in the degree of catheter obstruction. Only 100% obstructed catheter has no flow due to complete epoxy resin occlusion (*** *p* < 0.001). (**D**) Pressure measured in the silicone tubes instead of the phantom. The response time * to react to the given CSF input is 6 s. (* Time to reach maximum pressure). (** The pump was turned off beforehand to avoid sensor failure due to excessive pressure build-up in the silicone tube).

The internal pressure of the phantom model did not show a significant difference with varying degrees of catheter obstruction. Significant pressure elevation was found only when the completely obstructed catheters were assessed, relative to the partially occluded catheters (*** *p* < 0.001). There was a gradual, but not statistically significant change in intra-phantom pressure with varying degrees of catheter occlusion (Figure 2A). Pressure measured at the outflow of the catheters showed significant differences between 0% epoxy occlusion catheter (* *p* < 0.05) and all other partially occluded catheters, indicating that partial obstruction of the catheter significantly reduces the pressure at the outflow of the catheter. The 100% obstructed catheters had no pressure change at the distal end of the catheter (*** *p* < 0.001) as CSF flow through the catheter was completely blocked (Figure 2B). Flow measured at the catheter outflow showed no difference with various degrees of catheter obstruction (Figure 2C). This suggests that partial occlusion of the catheter may affect the outflow pressure (decreases pressure) at the distal end of the catheter without changing the catheter flow rate or quantity of CSF drainage.

The pressure measured from the occluded catheters inserted into the silicone tubing demonstrated differing properties compared to the catheters located within the ventricular phantom (Figure 2D). Based on the applied change in CSF input, both indicated pressure increased at ~0.07 psi, with an immediate pressure change in the silicone tubing and a gradual increase in the phantom model. There was also a significant difference in overall response time to the pressure change, with the silicone tubing reaching maximum pressure in 6 s and the phantom model reaching maximum pressure in >4 min. The pressure in the phantom thus more closely mimicked physiologic brain intraventricular pressure changes, as there was compliance in the phantom ventricle structure, but virtually none in the silicone catheter.

## 4. Discussion

Few models have been developed to elucidate the mechanisms underlying catheter obstruction; with most focusing on glial cell reaction or blood coagulation [10,11,12,13,14,15]. Many studies analyzed the characteristics of the catheter using simulation software [16,17,18], but very limited studies have been reported on CSF flow analysis in benchtop models. Most of these have focused on postoperative clinical data. In general, analysis using simulation only reflects the experimental environment determined by the values directly input by the user, so it may be more limited and inaccurate compared to the results in actual benchtop experiments. This study describes a 3D-printed phantom ventricular replicating system that assesses artificial catheter obstruction with several obvious limitations of conventional in-vitro catheter experiments. The phantom model was fabricated and designed to replicate the structural and compliant characteristics of a brain with hydrocephalus and as a result, exhibited different hydraulic properties than the experiments using solely silicone tubing.

The pressure characteristic measured in our phantom model shows a very gradual increase in pressure correlating with the infusion of CSF into the phantom. This was compared to pressure changes in a silicone tube, basically used as a control. The phantom exhibited higher flexibility (or a lower Young’s modulus) than silicone tubing, so the pressure change in the phantom is less responsive to the internal pressure build-up. This confirms that silicone tubing is more rigid than phantom walls. Although the Young’s modulus of the real brain is 1~4 kPa, which is lower than that of the phantom (1.5 Mpa), our phantom model shows that the high flexibility of the material allows a significant difference in the hydrodynamics of CSF flow compared to conventional in-vitro experiments.

Our model of phantom compliance likely mimics that in human brain ventricles. However, ventricle compliance is different in all patients, depending on their etiology of hydrocephalus. In our model, the phantom was compliant enough to tolerate an increase in the CSF input without a change in pressure, but only if there was patent CSF outflow. This might suggest that the ventricle in a patient with shunted hydrocephalus will not enlarge if at least one hole on the catheter is patent.

Our findings related to flow through the proximal catheter do not conclusively address shunt failure dynamics, as our study shows that CSF flow is maintained, even with near-complete catheter occlusion. This result is also consistent with the previous research results of Ginsberg et al. [19]. Our study also showed that the intra-catheter pressure required to maintain CSF flow was lower as more catheter holes were occluded. Additional studies will be needed to determine the extent of catheter occlusion before failure occurs. Our data indicate that a ventricular catheter that is almost entirely occluded will still have adequate CSF outflow. This may suggest that an initial shunt tap assessment performed as a workup of suspected shunt failure, could provide misleading results. Currently, the ability to extract CSF during a shunt tap is thought to be reflective of shunt patency. However, our results indicate that a partially obstructed catheter does not necessarily reduce CSF flow. Instead, it reduces the outflow pressure of the catheter with only a subtle increase in phantom ventricle pressure, suggesting that normal physiologic rates of CSF flow are maintained, even with only one patent hole in the catheter. Since increased ICP could be associated with an adequate flow rate despite a partially obstructed catheter, flow rate alone may not be the best indicator of catheter performance. As a result, the performance of an adequate shunt tap should not disregard the possibility of partial or even near complete proximal shunt failure, especially if clinical symptoms are present. A solitary patent catheter hole would more likely be occluded intermittently, than 24 patent holes on a catheter. This occlusion could be from tissue that acts as a “flap” to close the patent hole or even debris that occludes the hole and is washed down in the shunt tubing with normal CSF flow. These clinical situations might mimic intermittent shunt failure.

### Limitations

There are numerous limitations associated with this model, including the exclusion of astrocytes and choroid plexus from our experiment. Both have been implicated as the primary cellular drivers of proximal shunt occlusion, and there may be other cellular or physiological drivers contributing to shunt occlusion that were not present in our 3D printed phantom model. In order to produce a phantom to more closely reproduce physiologic hydrocephalus, a material similar to the brain in terms of flexibility and durability is required. For future studies, we will design various phantom shapes, volumes, and wall thicknesses, and analyze the Young’s modulus for a more detailed comparison. In addition, it is necessary to analyze more detailed hydrodynamic characteristics by measuring more parameters such as differential pressure and flow velocity at various locations (e.g., catheter inlet pressure, phantom wall pressure). Future experiments will be performed with a peristaltic pump to create pulsatile flow, generating a more physiologic CSF flow pattern.

## 5. Conclusions

In this novel phantom ventricular replicating system, partial occlusion of the catheter did not result in a decrease in the CSF flow rate but did decrease the outflow pressure in the ventricular catheter. There was no significant change in intra-phantom pressure, but rather only a gradual increase in pressure with partial occlusion of the catheters.
